# The impact of the COVID-19 pandemic on the global burden of type 2 diabetes: a study based on GBD 2021 data

**DOI:** 10.3389/fendo.2025.1600333

**Published:** 2025-10-15

**Authors:** Xiaoqin Chen, Hui Lin, Yinlian Wu, Mingfang Wang, Su Lin, Jiaofeng Huang

**Affiliations:** ^1^ Department of Infectious Disease, Second Hospital of Sanming, Sanming, Fujian, China; ^2^ Department of Emergency Intensive Care Unit, the First Affiliated Hospital, Fujian Medical University, Fuzhou, Fujian, China; ^3^ Department of Hepatology, Hepatology Research Institute, the First Affiliated Hospital, Fujian Medical University, Fuzhou, Fujian, China; ^4^ Fujian Clinical Research Center for Hepatopathy and Intestinal Diseases, Fuzhou, Fujian, China; ^5^ Department of Hepatology, National Regional Medical Center, Binhai Campus of the First Affiliated Hospital, Fujian Medical University, Fuzhou, Fujian, China

**Keywords:** Global Burden of Disease, type 2 diabetes, COVID-19, burden, epidemiology

## Abstract

**Background:**

This study aimed to comprehensively assess the impact of the coronavirus disease 2019 (COVID-19) pandemic on the global burden of type 2 diabetes mellitus (T2DM) using data from the Global Burden of Diseases (GBD) 2021.

**Methods:**

Age-standardized T2DM prevalence, incidence, mortality, and disability-adjusted life years (DALYs) were analyzed from Global Burden of Diseases (GBD) 2021. Pre-pandemic (2010–2019) and post-pandemic (2020–2021) periods were defined to capture pandemic onset, with 2020–2021 as the earliest available global post-pandemic data. Expected annual percentage changes (EAPC) were derived to assess the magnitude and direction of trends over the study period, adjusting for age, sex, and sociodemographic index. Post-pandemic projections to 2030 employed a Bayesian model with pre-/post-pandemic slope comparisons.

**Results:**

Global T2DM prevalence (ages ≥20) rose from 6,887.07 per 100,000 in 2010 to 9,545.42 per 100,000 in 2021. Post-pandemic acceleration was observed: EAPC increased from 2.90% (95% CI: 2.86–2.94) pre-pandemic to 3.52% (95% CI: 3.14–3.90) post-pandemic. Mortality and DALYs showed similar accelerations. Despite continued increases in incidence, mortality, and DALYs, the EAPC decreased in the post-pandemic period. Projections using pre-pandemic data (up to 2019) estimated type 2 diabetes prevalence at 10368.23 per 100,000 population by 2030. However, incorporating post-pandemic data (up to 2021) increased the projected 2030 prevalence to 10694.79 per 100,000, indicating a rise of 326.56 per 100,000 attributed to the pandemic’s impact. The prevalence, incidence, deaths, and DALYs of CKD due to T2DM all showed an upward trend from 2010 to 2021.

**Conclusions:**

A rapid increase in the burden of T2DM is found post-COVID-19 pandemic. Enhanced public health interventions are required for the prevention, screening, and management of diabetes.

## Introduction

Diabetes mellitus is a chronic metabolic disease characterized by insulin resistance and elevated blood glucose levels. The global epidemiology of diabetes has demonstrated a significant increase in its prevalence and incidence in recent decades, making it a major public health concern (1). According to estimates from the Global Burden of Diseases (GBD), high blood glucose is ranked as the fifth leading cause of the global disease burden, and diabetes itself was identified as the seventh leading cause of death globally in 2021 (2, 3). There are two major types of diabetes: type 1 and type 2. Type 2 diabetes mellitus (T2DM) is strongly correlated with obesity and sedentary lifestyles (4). The global population of individuals living with diabetes was approximately 529 million in 2021, and T2DM accounts for up to 96% of these cases (1). The detrimental effects of T2DM manifest in various forms, including kidney disease, cardiovascular disease, and neurological damage (5, 6).

Chronic kidney disease (CKD) due to T2DM, as one of the most common microvascular complications of diabetes and the leading cause of end-stage renal disease, has surpassed 107 million worldwide in 2021 (7, 8). Therefore, CKD due to T2DM is not only a key challenge in diabetes management but also a core issue in the global prevention and control of chronic non-communicable diseases and the improvement of public health outcomes.

Coronavirus disease 2019 (COVID-19) is an infectious disease caused by the severe acute respiratory syndrome coronavirus 2 (SARS-CoV-2) (9). The COVID-19 pandemic has had a profound global impact since its emergence in 2019. Public health measures, such as lockdowns and closures of recreational facilities during the pandemic, have led to reduced physical activity and increased sedentary behaviors, potentially exacerbating the risk of metabolic diseases, including T2DM. Studies have reported a higher incidence of obesity and metabolic syndrome following the COVID-19 outbreak (10, 11). Additionally, COVID-19 may worsen preexisting diabetes cases and increase the likelihood of new-onset diabetes (12). Furthermore, the diversion of healthcare resources to COVID-19 response efforts and restrictions on non-essential healthcare services may have exacerbated the challenges in diabetes management and prevention. Furthermore, beyond the indirect effects of lockdowns, SARS-CoV-2 infection itself has been directly implicated in worsening glucose metabolism and increasing diabetes risk, as a growing body of evidence suggests that COVID-19 may exacerbate pre-existing diabetes and contribute to new-onset diabetes (12–16). Large cohort studies and meta-analyses have reported that individuals who recovered from COVID-19 exhibit a significantly increased incidence of new-onset diabetes compared to those with non-COVID respiratory infections (17). Similarly, patients with pre-existing diabetes have shown worse glycemic control and higher rates of adverse outcomes post-COVID-19 infection (18).

Based on previous evidence, we hypothesized that the burden of T2DM may increase in the post-pandemic era. Thus, we utilized the Global Burden of Disease (GBD) 2021 dataset to analyze trends in the T2DM burden over the pre-pandemic decade and the subsequent two years. Specifically, our objectives were to analyze the global and regional prevalence, incidence, mortality, and disability-adjusted life-years (DALYs) associated with T2DM among individuals aged 20 years and older; to evaluate the disease burden attributable to T2DM-related CKD; and to project the future burden of T2DM by the year 2030.

## Methods

### Data source

This study utilized the GBD database, a comprehensive resource that quantifies health levels and trends worldwide. The GBD data were publicly accessible through the Institute for Health Metrics and Evaluation website (http://www.healthdata.org). The data download date for this study was June 24, 2024. The specific version of the GBD data employed in this study was GBD 2021, which includes the most recent updates after the COVID-19 pandemic. The study was restricted to individuals aged 20 years and older, as the GBD database does not include diabetes data for those under 15 years and provides limited data for the 15–19 age group, which could lead to inaccurate estimates of the overall disease burden.

### Definition

There is no universally defined starting point for the post-COVID era. COVID-19 began in 2019 and was declared a pandemic by the World Health Organization (WHO) on March 11, 2020. For this study, we defined the pre-pandemic era as the period before 2019 (specifically 2010-2019), and the post-pandemic period as the period after 2019, specifically 2020-2021. This timeframe was chosen because the updated GBD data only covers the period up to late 2021.

Disease burden indices, including the estimated rate and 95% uncertainty intervals (UI) of prevalence, incidence, death, and DALYs, were downloaded from the GBD system. The expected annual percentage change (EAPC) was used to summarize burden trends over time. The computation of the EAPC is grounded in a regression model formulated as follows: Y=α+βX+ϵ, where Y denotes the natural logarithm of the rate, X symbolizes the calendar year, α is a constant, β indicates positive or negative changing trends, and ϵ represents the error term (19). The EAPC was derived from the formula 100×(exp(β)−1), which provides a direct measure of the average annual change in the metric. The trends were interpreted as follows: a decrease if both the EAPC and its 95% confidence interval (CI) were ≤ 0; an increase if both were ≥ 0; and stability if neither condition was met. The EAPC from 2010 to 2019 indicates changes in the prevalence of type-2 diabetes during the pre-pandemic period, whereas the EAPC from 2020 to 2021 indicates changes during the post-pandemic period.

### Statistical analysis

Statistical analyses were performed using the R software version 4.3.3. Time trend graphs were generated to visually depict the disease incidence over the study period. Uncertainty intervals (UI) provided by GBD were used to assess variability in estimates. Missing data were handled through GBD’s modeling process, which uses spatiotemporal Gaussian process regression to impute and smooth estimates. Subgroup analyses stratified by sociodemographic index (SDI) quintiles and 204 countries were conducted to explore regional variations in T2DM trends. SDI is formulated based on three critical parameters: per capita income, average educational duration, and fertility rate, thereby providing a consolidated measure for socio-demographic evaluation. Prediction was performed using the Norpred package of the R software, which fits a series of Age-Period-Cohort models to the historical data. The core assumption of the selected model is the extrapolation of recent, stable trends in age-specific rates, period-specific deviations, and cohort-specific risks. The difference between the two estimates was considered statistically significant if the two confidence intervals did not overlap (20).

## Results

### Prevalence

**Figure 1:**
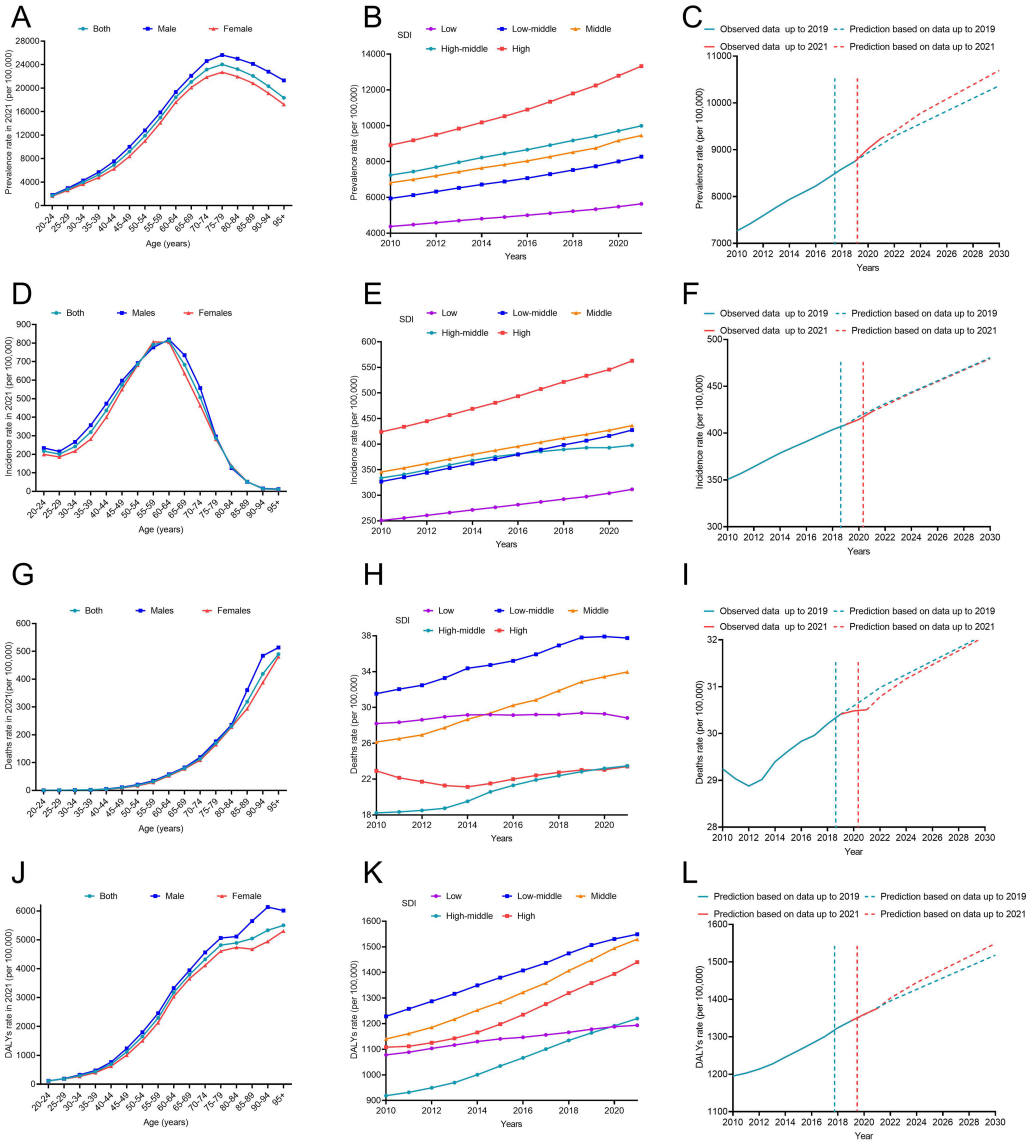
The disease burden of T2DM from 2010 to 2021 and its prediction to 2030. **(A)** Prevalence stratified by age in 2021; **(B)** Prevalence stratified by SDI regions from 2010 to 2021; **(C)** The prediction of prevalence to 2030; **(D)** Incidence stratified by age in 2021; **(E)** Incidence stratified by SDI regions from 2010 to 2021; **(F)** The prediction of incidence to 2030; **(G)** Deaths stratified by age in 2021; **(H)** Deaths stratified by SDI regions from 2010 to 2021; **(I)** The prediction of deaths to 2030; **(J)** DALYs stratified by age in 2021; **(K)** DALYs stratified by SDI regions from 2010 to 2021; **(L)** The prediction of DALYs to 2030. DALYs, disability-adjusted life years; SDI, sociodemographic index.

**Figure 2:**
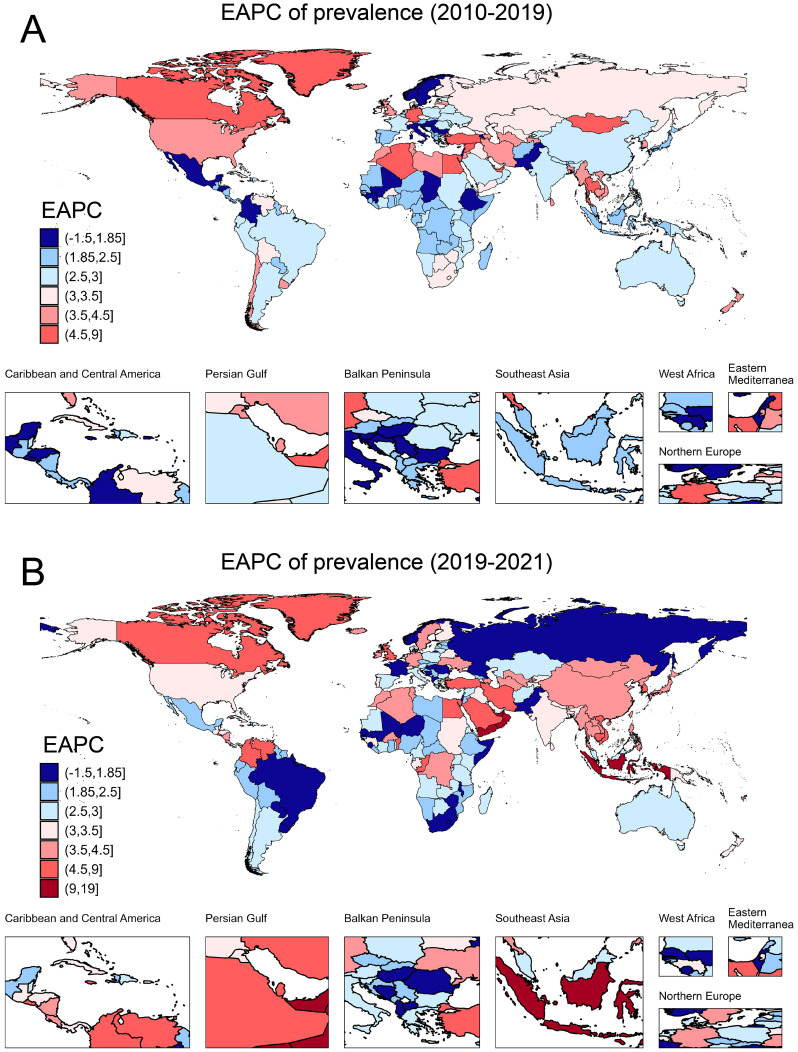
The EAPC of prevalence due to T2DM in 204 countries and territories. **(A)** The EAPC of prevalence from 2010 to 2019; **(B)** The EAPC of prevalence from 2020 to 2021. EAPC, estimated annual percentage change.

In 2021, approximately 501.66 million people worldwide were diagnosed with T2DM aged over 20 years. The prevalence has shown a continuous increase from 6887.07 per 100,000 in 2010 to 9545.42 per 100,000 in 2021 (**Table 1**). Prevalence increased with age and peaked in the 80-year-old group (**Figure 1A**). The global prevalence rate continued to increase from 2010 to 2021. The increasing trend of prevalence accelerated after the COVID-19 pandemic, with EAPC of 2.90% (2.86%–2.94%) before the pandemic and 3.52% (3.14%–3.90%) after the pandemic (**Table 1**). Overall, T2DM increased with increasing SDI levels; high SDI regions consistently had the highest prevalence of T2DM, followed by the high-middle SDI regions (**Figure 1B**). Notably, the rate of increase was more rapid in high SDI regions. Using pre-pandemic data (up to 2019) for prediction, the estimated prevalence of T2DM among the population aged over 20 years was projected to be 9113.14 per 100,000 in 2021 and 10368.23 per 100,000 in 2030. However, when using the post-pandemic data (up to 2021) for analysis, the predictive prevalence in 2030 would be 10694.79 per 100,000 population (326.56 per 100,100 population more), indicating a higher trend in the prevalence of diabetes after the onset of the COVID-19 pandemic (**Figure 1C**). Indonesia has experienced the most rapid increase in the prevalence of T2DM globally. From 2010 to 2019, the prevalence rose from 3513.75 per 100,000 population to 4285.44 per 100,000 population, and further spiked to 6054.97 per 100,000 population in 2021. The EAPC was 2.18% (2.12% to 2.24%) during 2010-2019, then increased to 18.87% (0.20%–41.00%) from 2019–2021 after the pandemic (**Table 2**). Similar trends were observed in Oman, Yemen, the United Arab Emirates, Canada, Colombia, the United Kingdom, Vietnam, and the Republic of Korea (**Table 2**; **Figures 2A, B**).

**Table 1:** The burden and EAPC of T2DM before and after the COVID-19 pandemic.

Valuables	Estimated rate (per 100,000)			EAPC (95% CI)	
	2010	2019	2021	2010-2019	2020-2021
Prevalence					
Total	6,887.07	8,907.30	9,545.42	2.9 (2.86 to 2.94)	3.52 (3.14 to 3.9)
Gender					
Male	7,180.42	9,291.49	9,948.03	2.9 (2.86 to 2.95)	3.47 (2.97 to 3.98)
Female	6,599.49	8,530.70	9,150.95	2.9 (2.86 to 2.93)	3.57 (3.33 to 3.81)
SDI					
Low	4,375.82	5,334.05	5,634.38	2.21 (2.16 to 2.26)	2.78 (2.75 to 2.8)
Low-middle	5,941.29	7,735.80	8,269.28	2.95 (2.9 to 3.01)	3.39 (3.33 to 3.45)
Middle	6,814.27	8,751.89	9,457.17	2.81 (2.77 to 2.85)	3.95 (2.98 to 4.93)
High-middle	7,240.60	9,413.22	9,994.84	2.98 (2.89 to 3.07)	3.04 (2.95 to 3.13)
High	8,915.25	12,246.35	13,324.03	3.6 (3.51 to 3.69)	4.31 (4.14 to 4.48)
Incidence					
Total	345.05	418.54	435.14	2.2 (2.12 to 2.28)	1.96 (1.61 to 2.32)
Gender					
Male	361.97	438.98	455.56	2.2 (2.12 to 2.27)	1.87 (1.5 to 2.24)
Female	328.46	398.50	415.13	2.2 (2.11 to 2.28)	2.06 (1.72 to 2.41)
SDI					
Low	250.85	297.40	311.63	1.93 (1.9 to 1.96)	2.36 (2.18 to 2.55)
Low-middle	326.86	406.71	427.51	2.47 (2.43 to 2.52)	2.53 (2.25 to 2.8)
Middle	345.50	419.14	436.14	2.19 (2.12 to 2.26)	2.01 (1.86 to 2.16)
High-middle	333.81	392.91	397.66	1.89 (1.65 to 2.13)	0.6 (-0.07 to 1.28)
High	423.96	533.50	562.77	2.63 (2.59 to 2.66)	2.71 (2.19 to 3.22)
Deaths					
Total	25.21	30.02	30.56	2.07 (1.88 to 2.27)	0.88 (0.75 to 1.02)
Gender					
Male	24.38	29.04	29.59	2.04 (1.85 to 2.23)	0.95 (0.57 to 1.32)
Female	26.03	30.99	31.51	2.1 (1.9 to 2.31)	0.83 (0.75 to 0.91)
SDI					
Low	28.20	29.39	28.83	0.43 (0.29 to 0.56)	-0.96 (-1.64 to -0.27)
Low-middle	31.54	37.81	37.75	2.02 (1.9 to 2.15)	-0.07 (-0.49 to 0.34)
Middle	26.13	32.87	33.96	2.65 (2.5 to 2.8)	1.65 (1.58 to 1.72)
High-middle	18.23	22.83	23.48	2.89 (2.49 to 3.29)	1.41 (1.19 to 1.64)
High	22.92	23.01	23.40	0.3 (-0.37 to 0.98)	0.84 (0 to 1.69)
DALYs					
Total	1,101.51	1,365.02	1,426.37	2.47 (2.34 to 2.59)	2.22 (2.04 to 2.4)
Gender					
Male	1,127.98	1,398.50	1,461.33	2.45 (2.33 to 2.58)	2.22 (1.86 to 2.58)
Female	1,075.56	1,332.19	1,392.12	2.48 (2.35 to 2.61)	2.22 (2.22 to 2.23)
SDI					
Low	1,077.93	1,178.18	1,193.61	0.98 (0.9 to 1.05)	0.65 (0.43 to 0.88)
Low-middle	1,228.50	1,506.79	1,548.90	2.28 (2.25 to 2.31)	1.39 (1.17 to 1.61)
Middle	1,140.24	1,448.62	1,529.19	2.73 (2.6 to 2.87)	2.74 (2.25 to 3.24)
High-middle	918.76	1,164.00	1,219.86	2.8 (2.61 to 3)	2.37 (2.34 to 2.4)
High	1,107.95	1,358.43	1,439.97	2.41 (2.06 to 2.75)	2.96 (2.59 to 3.32)

COVID-2019, coronavirus disease 2019; SDI, socio-demographic index; DALYs, disability-adjusted life years; EAPC, Estimated Annual Percentage Change; CI, confidence interval.

**Table 2:** The top 10 countries with rapid disease burden growth after the COVID-19 pandemic.

Countries	Estimated rate (per 100,000)			EAPC (95% CI)	
	2010	2019	2021	2010-2019	2020-2021
Prevalence					
Indonesia	3,513.75	4,285.44	6,054.97	2.18 (2.12 to 2.24)	18.87 (0.20 to 41.00)
Oman	4,905.39	6,431.96	7,998.84	2.97 (2.66 to 3.29)	11.52 (8.8 to 14.30)
Yemen	3,748.70	5,062.71	6,187.17	3.38 (3.28 to 3.48)	10.55 (1.41 to 20.51)
United Arab Emirates	3,892.68	8,113.57	9,753.16	8.53 (8.32 to 8.74)	9.64 (8.56 to 10.73)
Canada	6,959.10	10,396.31	12,319.00	4.5 (4.43 to 4.57)	8.85 (7.93 to 9.79)
Greenland	2,687.14	5,238.86	6,076.09	7.77 (7.62 to 7.92)	7.69 (7.60 to 7.79)
Colombia	7,040.59	8,171.00	9,469.96	1.7 (1.44 to 1.96)	7.66 (2.15 to 13.46)
United Kingdom	8,591.20	12,054.21	13,737.11	3.87 (3.36 to 4.37)	6.75 (2.47 to 11.22)
Viet Nam	3,878.70	5,512.55	6,271.20	3.99 (3.72 to 4.27)	6.66 (4.59 to 8.77)
Republic of Korea	11,592.23	17,290.95	19,440.50	4.47 (4.06 to 4.88)	6.03 (6.00 to 6.07)
Incidence					
Oman	367.39	475.76	568.73	2.86 (2.51 to 3.21)	9.34 (5.9 to 12.88)
United Arab Emirates	348.98	687.37	810.52	7.93 (7.81 to 8.04)	8.59 (7.18 to 10.01)
Colombia	358.18	385.03	435.04	0.84 (0.51 to 1.16)	6.30 (1.63 to 11.18)
Greenland	189.70	322.10	362.83	6.15 (6 to 6.3)	6.13 (5.69 to 6.58)
Yemen	250.83	332.50	374.43	3.18 (3.08 to 3.28)	6.12 (2.31 to 10.07)
Viet Nam	271.96	388.94	436.26	4.08 (3.79 to 4.37)	5.91 (4.92 to 6.90)
Benin	278.29	330.05	369.11	1.85 (1.77 to 1.93)	5.75 (3.46 to 8.10)
United Kingdom	316.13	413.29	459.54	3.05 (2.62 to 3.48)	5.45 (1.61 to 9.43)
Canada	422.79	554.18	615.17	3.02 (2.92 to 3.12)	5.36 (4.97 to 5.75)
Qatar	641.54	846.62	939.73	3.21 (2.88 to 3.54)	5.36 (4.01 to 6.72)
Deaths					
Venezuela (Bolivarian Republic of)	36.32	60.62	69.29	5.82 (5.21 to 6.43)	6.92 (5.09 to 8.78)
Bahrain	41.92	52.55	58.85	2.63 (2.17 to 3.08)	5.83 (5.76 to 5.9)
Saint Lucia	80.23	88.59	98.25	1.33 (0.28 to 2.38)	5.31 (5.03 to 5.6)
Republic of Korea	27.52	22.57	24.43	-2.45 (-2.8 to -2.1)	4.05 (0.84 to 7.37)
Iraq	31.85	37.04	40.09	1.45 (0.73 to 2.17)	4.04 (-0.92 to 9.24)
Northern Mariana Islands	66.46	78.49	84.83	1.54 (1.22 to 1.86)	3.96 (2.38 to 5.56)
Jordan	30.78	27.51	29.62	-1.68 (-2.06 to -1.3)	3.76 (2.65 to 4.89)
Thailand	31.30	42.36	45.53	3.42 (2.84 to 4)	3.67 (3.59 to 3.75)
Malta	33.11	38.46	40.95	3.09 (1.64 to 4.57)	3.19 (-4.81 to 11.86)
Puerto Rico	113.57	111.79	118.88	-0.54 (-1.37 to 0.3)	3.12 (1.62 to 4.64)
DALYs					
Venezuela (Bolivarian Republic of)	1,519.81	2,290.59	2,612.50	4.49 (4.1 to 4.89)	6.8 (4.19 to 9.47)
Indonesia	1,011.77	1,211.39	1,377.19	2.02 (2 to 2.04)	6.62 (0.81 to 12.78)
Yemen	525.88	653.93	734.82	2.4 (2.25 to 2.54)	6 (1.33 to 10.89)
Colombia	1,064.22	1,170.76	1,315.09	1.08 (0.89 to 1.26)	5.98 (1.77 to 10.38)
Bahrain	1,791.27	2,474.16	2,762.86	3.69 (3.58 to 3.82)	5.67 (5.36 to 5.99)
United Arab Emirates	463.53	882.26	979.63	7.21 (6.9 to 7.51)	5.37 (0.91 to 10.03)
Republic of Korea	1,423.80	1,727.78	1,918.19	2.02 (1.44 to 2.6)	5.37 (4.52 to 6.22)
Canada	979.22	1,176.59	1,304.91	2.31 (1.69 to 2.95)	5.31 (4.94 to 5.68)
United Kingdom	746.70	988.01	1,090.38	3.31 (2.71 to 3.91)	5.05 (1.1 to 9.16)
Singapore	903.58	1,066.16	1,166.91	1.92 (1.57 to 2.26)	4.62 (4.27 to 4.96)

COVID-2019, coronavirus disease 2019; DALYs, disability-adjusted life years; EAPC, Estimated Annual Percentage Change; CI, confidence interval.

### Incidence

**Figure 1D** shows the sex-age-stratified incidence in 2021, with no difference between sexes, and the highest incidence was observed in the 60–65 age group. The incidence continued to increase over time, and increased EAPCs were observed in almost all SDI regions except for the high-middle SDI region (**Figure 1E**), where the EAPC decreased from 1.89% (1.65%–2.13%) pre-pandemic to 0.60% (-0.07%–1.28%) post-pandemic (**Table 1**). The predicted incidence of T2DM in 2030 was similar with and without the COVID-19 pandemic (**Figure 1F**). Countries such as Oman, the United Arab Emirates, Colombia, Greenland, Yemen, Vietnam, Benin, the United Kingdom, Canada, and Qatar exhibited the most rapid increase in the incidence of T2DM post-pandemic (**Table 2**; **Supplementary Figures 1A, B**).

### Deaths

In 2021, the number of deaths due to T2DM began to rise in the 50 age group and sharply increased with age. No significant difference was observed between males and females before the age of 85. After the age threshold, the death rate due to T2DM surpassed that of females by 2021(**Figure 1G**). The global death rate continued to increase from 2010 to 2021, but the rate of increase slowed down, with an EAPC of 2.07% (1.88% to 2.27%) from 2010 to 2019 and 0.88% (0.75% to 1.02%) from 2019 to 2021(**Table 1**). The low–middle SDI regions consistently had the highest mortality rates. The middle SDI region had the second-highest mortality rate in 2021 (**Figure 1H**). The results of the prediction showed that the presence of COVID-19 led to a reduction in T2DM-related deaths during the first two years following the onset of the pandemic. However, this effect weakened over time (**Figure 1I**). In some countries, the death rate due to T2DM is still on the rise, such as the Bolivarian Republic of Venezuela, Bahrain, Saint Lucia, and the Republic of Korea (**Supplementary Figures 1C, D**).

### DALYs

Despite the decline in global death rates, the number of DALYs due to T2DM has continued to increase from 1101.51 per 100,000 in 2010 to 1426.37 per 100,000 in 2021 (**Table 1**). The DALY increased with age, and there was no significant sex difference in DALYs before the age of 85 years (**Figure 1J**). The high SDI regions exhibited particularly high DALY rates (**Figure 1K**). **Figure 1L** shows a higher predictive DALYs for 2030, after the pandemic. The Bolivarian Republic of Venezuela, Indonesia, Yemen, Colombia, and Bahrain were identified as the five countries with the highest rates of increase in DALYs (**Table 2**; **Supplementary Figures 1E, F**).

### Burden of CKD due to T2DM

**Figure 3:**
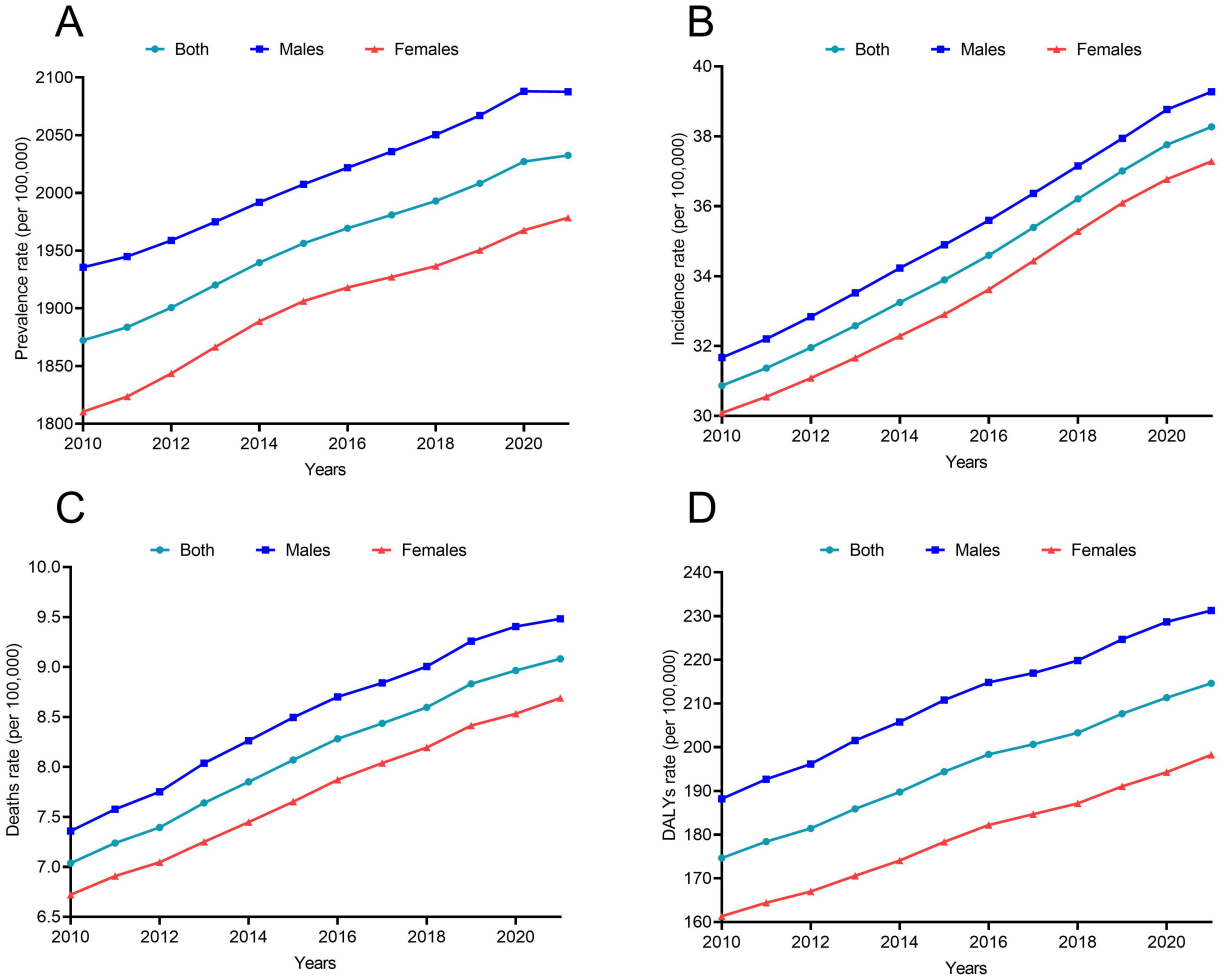
The disease burden of CKD due to T2DM from 2010 to 2021 **(A)** Prevalence; **(B)** Incidence; **(C)** Deaths; **(D)** DALYs.

While the prevalence of CKD due to T2DM continues to increase from 2010 to 2021 (**Figure 3A**), the incidence of CKD continues to rise from 30.87 per 100,000 in 2010 to 38.27 per 100,000 in 2021 (**Supplementary Table 1**; **Figure 3B**). The death and DALYs rates of CKD due to T2DM also showed an upward trend (**Figures 3C, D**), although at a reduced rate post-COVID-19. The EAPC of DALYs attributed to diabetes-related CKD were 1.96% (1.85% to 2.07%) before the pandemic and 1.65% (1.54% to 1.76%) after the pandemic.

## Discussion

This study is the first systematic analysis to use the GBD 2021 dataset to estimate the T2DM burden following the COVID-19 pandemic in people aged over 20 years. A notable increase in the prevalence of diabetes was observed in the post-pandemic era.

Our analysis of GBD 2021 data indicates that the global burden of T2DM not only continued its long-term increasing trend but also accelerated in the post-pandemic era. This acceleration may be attributed to a confluence of direct and indirect pathways associated with the COVID-19 pandemic, which collectively exacerbated the global diabetes landscape. The direct biological effects of SARS-CoV-2 infection appear to play a significant role. Evidence suggests that the virus can directly infect pancreatic β-cells via ACE2 receptors, impairing insulin secretion and potentially accelerating β-cell apoptosis (21). Furthermore, the systemic inflammatory response and stress hyperglycemia during infection, compounded by the release of counter-regulatory hormones and the use of corticosteroids in treatment, can promote significant insulin resistance and hyperglycemia (22–24). These mechanisms are supported by large-scale clinical studies reporting a substantially higher incidence of new-onset diabetes among COVID-19 survivors compared to non-infected controls (25, 26), and are further reinforced by genetic evidence from Mendelian randomization studies indicating a bidirectional relationship between genetic susceptibility to COVID-19 and T2DM risk (27). Simultaneously, the indirect consequences of pandemic policies profoundly influenced population-level risk factors. Lockdowns and social restrictions led to widespread behavioral changes, including reduced physical activity (28–30) and increased weight gain (28, 29), which exacerbated known risk factors for T2DM such as overweight, obesity, poor diet, and physical inactivity (31). These changes adversely affected glycemic control in both the general population (32) and existing T2DM patients (32, 33), creating a conducive environment for diabetes progression and new cases. Therefore, the observed increase in global T2DM burden is not attributable to a single cause but rather results from the synergistic interaction of viral pathogenesis and pandemic-induced societal changes. This integrated explanation underscores the multifaceted impact of COVID-19 on diabetes dynamics, illuminating how biological and environmental factors collectively drove the accelerated disease burden.

A notable shift was observed following the COVID-19 pandemic: although the incidence, mortality, and DALYs related to T2DM continued to increase, their rates of growth slowed, contrasting sharply with the accelerated rise in prevalence. This pattern may be partially attributable to disruptions in healthcare access and documentation during the pandemic, which likely compromised the reporting of new T2DM cases. Furthermore, the observed attenuation in mortality growth may reflect misclassification of cause of death among diabetic patients—a population at elevated risk of severe COVID-19 outcomes (34). In cases where diabetic individuals contracted COVID-19 and died, the primary cause of death may have been assigned to COVID-19 rather than diabetes, leading to potential underestimation of diabetes-related mortality in official statistics. Thus, the apparent moderation in mortality and DALY growth should not be misinterpreted as a genuine reduction in the disease burden of diabetes, particularly given the concurrent increase in prevalence.

In high-SDI regions (e.g., Canada, the United Kingdom), T2DM prevalence increased markedly post-pandemic, yet mortality declined. Modernized clinical protocols emphasizing early intensive management may mitigate fatal outcomes despite growing disease burden. National diabetes prevention programs (e.g., the UK’s NHS Digital Weight Management Programme) achieved sustained engagement through digital modalities during lockdowns (35). These interventions likely accelerated case detection while reinforcing preventive messaging. Sodium-glucose co-transporter 2 inhibitors and glucagon-like peptide-1 receptor agonists are widely used (36), and these drugs exhibit cardiovascular protective effects beyond blood glucose control. Mortality declines could partially reflect competing risks – reduced physical activity during lockdowns theoretically increased obesity-driven incidence, but paradoxically lowered transport accidents/occupational injuries.

Our study also revealed that although the prevalence and incidence of T2DM in Indonesia and other comparable regions did not show a significant increase following the COVID-19 pandemic, there was a marked rise in DALYs attributable to T2DM. We propose that in low SDI regions such as Indonesia, pandemic-related constraints on healthcare resources severely disrupted routine diabetes screening, diagnosis, and chronic disease management (37). These disruptions likely led to considerable underdiagnosis and suboptimal management of T2DM. As a consequence, many cases may have only been identified at advanced stages, resulting not only in substantially higher medical costs but also in an increased risk of long-term complications and sequelae for patients (38, 39). Furthermore, although Oman and the United Arab Emirates ranked among the top countries in terms of T2DM prevalence and incidence growth, they did not feature prominently in mortality rankings. This may reflect the successful implementation of national diabetes management programs, early intervention strategies, and high-quality healthcare services that effectively reduce case fatality rates. It is also possible that these countries have younger diabetic populations or better-controlled comorbidities, which could attenuate mortality risk despite high disease burden (40).

WHO established the first global target for diabetes mellitus in 2022, highlighting the urgent need to address this growing health challenge (41). Given the substantial and growing number of COVID-19 cases, their impact on diabetes is likely to result in a significant population-level burden. In 2014, the United Kingdom launched the Diabetes Prevention Program to mitigate the risk of diabetes. The initial outcomes of this program in 2018 revealed stable or declining prevalence rates of T2DM, with expectations of further decline in subsequent years (42). However, the unexpected acceleration in the post-pandemic diabetes burden in the United Kingdom, as highlighted by this study, suggests the need for ongoing assessment and adaptation of previous strategies. Several measures can be taken to mitigate the burden of diabetes in the post-pandemic era. These include educating and screening patients with SARS-CoV-2 infection for diabetes, enhancing the healthcare system capacity to ensure ongoing monitoring and treatment for individuals with diabetes, and implementing policies that advocate healthy lifestyle choices, such as dietary habits and physical exercise, to mitigate diabetes risk.

This study has several limitations. First, we focused solely on the burden of T2DM, as it accounts for up to 96% of all diabetes cases (1); however, this approach may not fully capture the impact on other forms of diabetes. Second, the analysis is limited to data up to 2021 due to GBD data availability. The pandemic’s evolving nature, including vaccinations and new variants beyond 2021, may influence long-term trends and should be investigated in future updates. Third, shifts in health priorities and public health efforts during the pandemic may have disrupted disease surveillance and reporting systems, potentially leading to delays and underreporting. While the GBD provides comprehensive global estimates, it relies on modeled data that may be subject to regional heterogeneity, reporting delays, and methodological assumptions. Additionally, the GBD modeling approach may incorporate certain biases and uncertainties, and regional heterogeneity in data availability and quality could affect the generalizability of our findings. Finally, due to data lag and the rapidly changing context of the pandemic, the long-term health effects of COVID-19 require continued monitoring and accumulation of more comprehensive data.

In conclusion, the global burden of T2DM, particularly its rising prevalence and DALYs, has escalated rapidly in the wake of the pandemic. Addressing this trend demands urgent implementation of evidence-based strategies for both management and prevention. These should include individual-level interventions—such as glycemic monitoring and lifestyle modification programs—as well as broader policy measures, for example, sugar taxes, public health education campaigns, and integrated primary care initiatives aimed at early detection and multidisciplinary support. Learning from successful models globally will be essential to designing effective, context-sensitive responses.

## Data Availability

The raw data supporting the conclusions of this article will be made available by the authors, without undue reservation.

## Supplementary material

Supplementary Figure 1 The EAPC of incidence, deaths, and DALYs due to T2DM in 204 countries and territories **(A)** The EAPC of incidence from 2010 to 2019; **(B)** The EAPC of incidence from 2020 to 2021; **(C)** The EAPC of deaths from 2010 to 2019; **(D)** The EAPC of deaths from 2020 to 2021; **(E)**The EAPC of DALYs from 2010 to 2019; **(F)** The EAPC of DALYs from 2020 to 2021. EAPC, estimated annual percentage change.
