# Supplementary figures and images for: The impact of the COVID-19 pandemic on the global burden of type 2 diabetes: a study based on GBD 2021 data
Source: Front Endocrinol (Lausanne). 2025 Oct 15;16:1600333. doi: 10.3389/fendo.2025.1600333 (PMC12568351; doi:10.3389/fendo.2025.1600333)

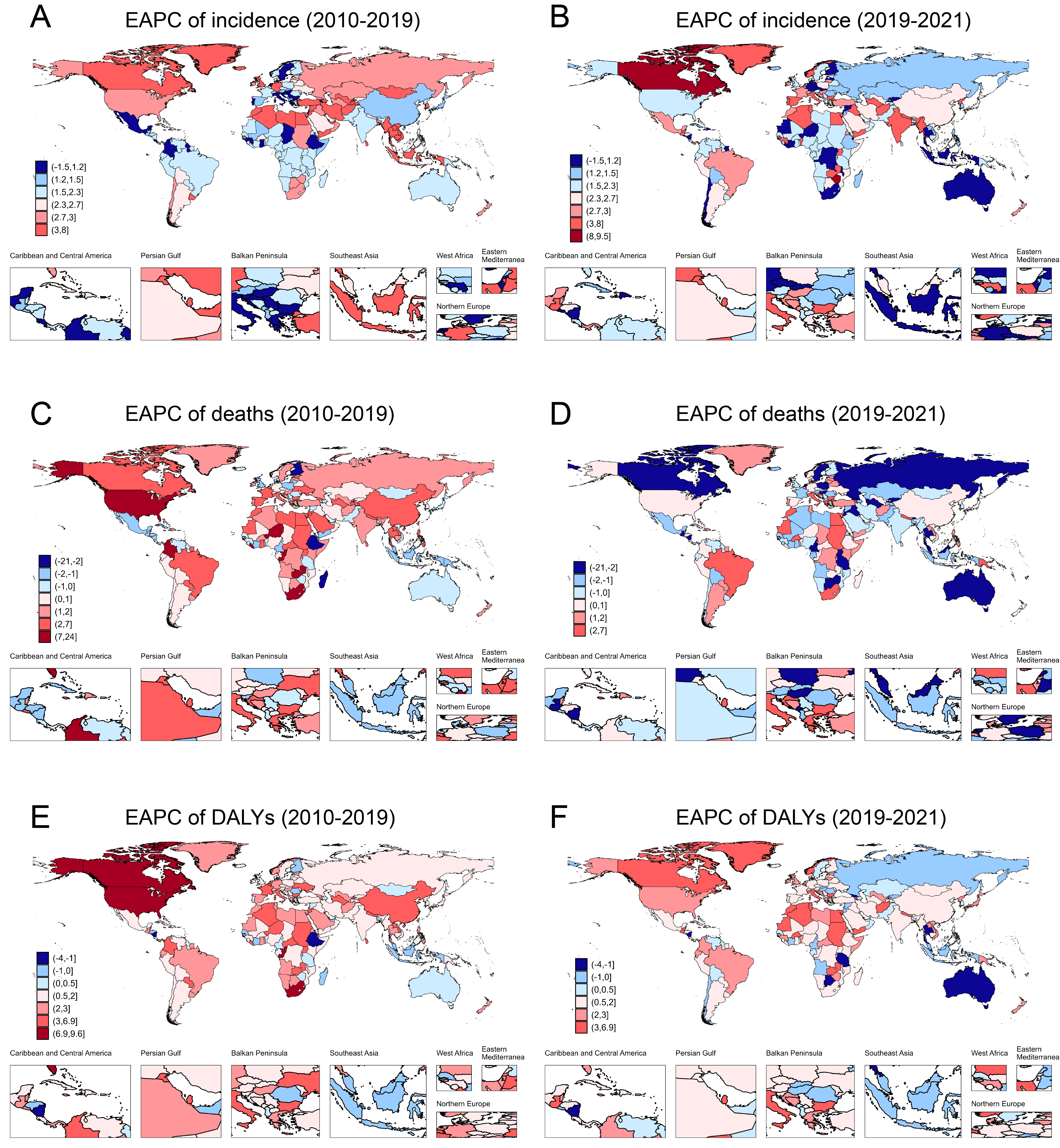

Supplement: Supplementary Figure 1 — The EAPC of incidence, deaths, and DALYs due to T2DM in 204 countries and territories (A) The EAPC of incidence from 2010 to 2019; (B) The EAPC of incidence from 2020 to 2021; (C) The EAPC of deaths from 2010 to 2019; (D) The EAPC of deaths from 2020 to 2021; (E)The EAPC of DALYs from 2010 to 2019; (F) The EAPC of DALYs from 2020 to 2021. EAPC, estimated annual percentage change. [file Image1.jpeg]
