# Supplementary material for: The impact of the COVID-19 pandemic on the global burden of type 2 diabetes: a study based on GBD 2021 data
Source: Front Endocrinol (Lausanne). 2025 Oct 15;16:1600333. doi: 10.3389/fendo.2025.1600333 (PMC12568351; doi:10.3389/fendo.2025.1600333)
Supplement: Supplementary file 2 [file Table1.docx]

Supplementary Table 1 The burden and EAPC of CKD due to type 2 diabetes before and after the COVID-19 pandemic

| Valuables | Estimated rate (per 100,000) | | | EAPC (95% CI) | |
| --- | --- | --- | --- | --- | --- |
|  | 2010 | 2019 | 2021 | 2010-2019 | 2020-2021 |
| **Prevalence** |  |  |  |  |  |
| **Total** | 1,872.38 | 2,008.18 | 2,032.46 | 0.8 (0.75 to 0.85) | 0.6 (0.22 to 0.99) |
| **Gender** |  |  |  |  |  |
| Male | 1,935.57 | 2,067.07 | 2,087.58 | 0.75 (0.73 to 0.77) | 0.49 (-0.09 to 1.08) |
| Female | 1,810.43 | 1,950.46 | 1,978.46 | 0.85 (0.76 to 0.95) | 0.72 (0.53 to 0.9) |
| **SDI** |  |  |  |  |  |
| Low | 1,525.09 | 1,519.56 | 1,503.45 | -0.06 (-0.26 to 0.13) | -0.53 (-1.03 to -0.03) |
| Low-middle | 1,966.16 | 2,025.41 | 2,028.85 | 0.3 (0.09 to 0.52) | 0.08 (-0.85 to 1.03) |
| Middle | 1,887.80 | 2,081.16 | 2,129.43 | 1.13 (0.97 to 1.28) | 1.15 (0.54 to 1.77) |
| High-middle | 1,876.71 | 2,050.55 | 2,098.68 | 1.04 (0.78 to 1.3) | 1.17 (1.03 to 1.3) |
| High | 1,900.12 | 2,082.41 | 2,097.20 | 1.05 (1.02 to 1.08) | 0.35 (-0.19 to 0.91) |
|  |  |  |  |  |  |
| **Incidence** |  |  |  |  |  |
| **Total** | 30.87 | 37.01 | 38.27 | 2.05 (1.98 to 2.11) | 1.69 (1.3 to 2.08) |
| **Gender** |  |  |  |  |  |
| Male | 31.67 | 37.94 | 39.27 | 2.04 (2.01 to 2.08) | 1.74 (1.25 to 2.23) |
| Female | 30.09 | 36.09 | 37.29 | 2.06 (1.95 to 2.16) | 1.64 (1.36 to 1.92) |
| **SDI** |  |  |  |  |  |
| Low | 12.93 | 14.23 | 14.47 | 1.06 (1.04 to 1.09) | 0.84 (0.18 to 1.51) |
| Low-middle | 21.71 | 24.99 | 25.75 | 1.57 (1.52 to 1.63) | 1.51 (0.96 to 2.06) |
| Middle | 27.46 | 35.27 | 37.37 | 2.84 (2.81 to 2.88) | 2.93 (2.64 to 3.23) |
| High-middle | 30.54 | 38.47 | 40.38 | 2.61 (2.52 to 2.71) | 2.45 (2.28 to 2.63) |
| High | 56.88 | 67.77 | 69.11 | 1.98 (1.75 to 2.2) | 0.99 (0.25 to 1.73) |
|  |  |  |  |  |  |
| **Deaths** |  |  |  |  |  |
| **Total** | 7.04 | 8.83 | 9.08 | 2.56 (2.45 to 2.68) | 1.4 (1.29 to 1.51) |
| **Gender** |  |  |  |  |  |
| Male | 7.36 | 9.26 | 9.48 | 2.58 (2.42 to 2.74) | 1.2 (0.76 to 1.64) |
| Female | 6.72 | 8.41 | 8.69 | 2.55 (2.47 to 2.62) | 1.62 (1.37 to 1.87) |
| **SDI** |  |  |  |  |  |
| Low | 5.27 | 5.63 | 5.53 | 0.74 (0.51 to 0.97) | -0.91 (-1.84 to 0.03) |
| Low-middle | 5.89 | 7.11 | 7.12 | 2.13 (1.92 to 2.35) | 0.06 (-0.57 to 0.69) |
| Middle | 8.38 | 10.31 | 10.72 | 2.35 (2.25 to 2.45) | 1.98 (1.74 to 2.22) |
| High-middle | 5.58 | 6.80 | 7.12 | 2.32 (2.17 to 2.47) | 2.31 (2.26 to 2.37) |
| High | 8.47 | 12.45 | 12.95 | 4.27 (3.71 to 4.83) | 2 (1.3 to 2.71) |
|  |  |  |  |  |  |
| **DALYs** |  |  |  |  |  |
| **Total** | 174.64 | 207.69 | 214.60 | 1.96 (1.85 to 2.07) | 1.65 (1.54 to 1.76) |
| **Gender** |  |  |  |  |  |
| Male | 188.20 | 224.67 | 231.29 | 1.98 (1.84 to 2.12) | 1.46 (1.1 to 1.83) |
| Female | 161.34 | 191.04 | 198.25 | 1.93 (1.84 to 2.01) | 1.87 (1.68 to 2.06) |
| **SDI** |  |  |  |  |  |
| Low | 137.28 | 142.96 | 142.75 | 0.44 (0.3 to 0.57) | -0.07 (-0.74 to 0.6) |
| Low-middle | 161.87 | 187.16 | 190.37 | 1.63 (1.47 to 1.79) | 0.85 (0.17 to 1.54) |
| Middle | 210.91 | 248.96 | 260.35 | 1.89 (1.79 to 1.98) | 2.26 (1.98 to 2.55) |
| High-middle | 139.29 | 160.34 | 167.75 | 1.63 (1.53 to 1.74) | 2.29 (2.05 to 2.52) |
| High | 182.41 | 247.30 | 255.70 | 3.38 (2.76 to 4) | 1.68 (0.91 to 2.46) |

**Abbreviations:** COVID-2019, coronavirus disease 2019; SDI, socio-demographic index; DALYs, disability-adjusted life years; EAPC, Estimated Annual Percentage Change; CI, confidence interval;.
